# Prediction of hypertension based on the genetic analysis of longitudinal phenotypes: a comparison of different modeling approaches for the binary trait of hypertension

**DOI:** 10.1186/1753-6561-8-S1-S78

**Published:** 2014-06-17

**Authors:** Yun-Hee Choi, Rafiqul Chowdhury, Balakumar Swaminathan

**Affiliations:** 1 Department of Epidemiology and Biostatistics, Schulich School of Medicine and Dentistry, Western University, 1151 Richmond Street, London, Ontario, Canada N6A5C1

## Abstract

For the analysis of the longitudinal hypertension family data, we focused on modeling binary traits of hypertension measured repeatedly over time. Our primary objective is to examine predictive abilities of longitudinal models for genetic associations. We first identified single-nucleotide polymorphisms (SNPs) associated with any occurrence of hypertension over the study period to set up covariates for the longitudinal analysis. Then, we proceeded to the longitudinal analysis of the repeated measures of binary hypertension with covariates including SNPs by accounting for correlations arising from repeated outcomes and among family members.

We examined two popular models for longitudinal binary outcomes: (a) a marginal model based on the generalized estimating equations, and (b) a conditional model based on the logistic random effect model. The effects of risk factors associated with repeated hypertensions were compared for these two models and their prediction abilities were assessed with and without genetic information.

Based on both approaches, we found a significant interaction effect between age and gender where males were at higher risk of hypertension before age 35 years, but after age 35 years, women were at higher risk. Moreover, the SNPs were significantly associated with hypertension after adjusting for age, gender, and smoking status. The SNPs contributed more to predict hypertension in the marginal model than in the conditional model. There was substantial correlation among repeated measures of hypertension, implying that hypertension was considerably correlated with previous experience of hypertension. The conditional model performed better for predicting the future hypertension status of individuals.

## Background

Hypertension is a chronic condition resulting from high blood pressure in the arteries during circulation. Clinically, a person is said to be hypertensive if the individual's systolic blood pressure (SBP) is greater than 140 mm Hg or diastolic blood pressure (DBP) is greater than 90 mm Hg. With advances in genome-wide association studies, several researchers have also investigated the role of genes in this disease [1,2]. It is essential to control hypertension in order to avoid consequences like cardiovascular diseases, stroke, and heart and kidney failure.

The San Antonio Family Study data for Genetic Analysis Workshop 18 (GAW18) contain up to 4 longitudinal measures of SBP and DBP along with their background and genetic information for a total of 932 individuals from 20 Mexican American families. In the analysis of the longitudinal hypertension family data, we focus on modeling longitudinal binary traits of hypertension, defined by SBP >140 mm Hg, DBP >90 mm Hg or use of antihypertensive medication, by taking into account correlations arising from repeated outcomes and among family members while controlling for covariates such as age, gender, smoking status, and genetic polymorphisms.

Our primary objective is to examine predictive abilities of longitudinal models with inclusion of genetic information. In the first step, we identify important single-nucleotide polymorphisms (SNPs) associated with any occurrence of hypertension over the study period in order to set up covariates for the longitudinal analysis. The selection of SNPs is based on chromosome 3 only. Then we proceed to the longitudinal analysis of repeated measures of hypertension with covariates, including SNPs that already identified. We examined two popular modeling approaches for longitudinal binary outcomes: the marginal model (population-average) and the conditional model (subject-specific). The effects of the risk factors associated with repeated hypertension from the two models were compared and their prediction abilities were assessed with and without genetic information using the areas under the receiver operating characteristic curve.

## Methods

### Selection of associated SNPs

The SNP selection was performed based on the Cox proportional hazards (PH) model [3,4] using time to first hypertension, and on the logistic model using any occurrence of hypertension over repeated measurements as a binary outcome, controlling for covariates of interest such as age, gender, and smoking status. The Cox PH model was fitted for each SNP with frailty, a random effect, to account for familial correlation,

(1) $$\lambda_{\text{ij}}\text{(t)}=\lambda_{0}\text{(t) exp}\;(\beta {\text{X}}_{\text{ij}}+\alpha \text{SNP}+{\text{b}}_{\text{j}}\text{)}$$

where λ_ij_(t) is the hazard function of individual *i *in family *j*, λ_0_(t) is the baseline hazard function, b_j _is the random effect for family *j*, and X_ij _is the vector of covariates for the fixed effects. Each SNP entered into the model was assumed to be codominant (TT, TC, CC) as the inheritance mode is not known. Similarly, the logistic model was fitted for each SNP for the binary hypertension outcome, defined as 1 if one has ever experienced hypertension over the study period and 0 otherwise, with a random intercept to account for familial correlation. These two modeling approaches were employed to validate the choice of SNPs for the following longitudinal models.

### Longitudinal models

We considered the two most commonly used modeling strategies for analyzing correlated data from longitudinal study: *marginal *and *conditional *models, also known as population-averaged and subject-specific approaches, respectively. To accommodate the dependence among longitudinal repeated outcomes, the marginal approach directly describes marginal means along with a prespecified working correlation structure as nuisance parameters, whereas the conditional approach describes an individual response conditioning on the unobserved values of random effects. The generalized estimating equations (GEEs) method by Liang and Zeger [5] was used to estimate population-average effects in the marginal model, whereas the logistic random effect model [6] was used to provide subject-specific effects in the conditional model.

#### Marginal model (population-averaged model)

Let y_ij _represent the occurrence of hypertension (binary trait) for individual *i *at time *j*. Then, the marginal logistic model for hypertension can be expressed as

(2) $$\text{logit P(}{\text{y}}_{\text{ij}}=\text{1};\;{\text{X}}_{\text{ij}}\text{)}=\beta_{0}+\beta_{\text{1}}\;\text{ag}{\text{e}}_{\text{ij}}+\beta_{\text{2}}\;\text{se}{\text{x}}_{\text{i}}+\beta_{\text{3}}\;\text{se}{\text{x}}_{\text{i}}\;\text{x ag}{\text{e}}_{\text{ij}}+\beta_{\text{4}}\;\text{smok}{\text{e}}_{\text{ij}}+\beta_{\text{5}}\;\text{SN}{\text{P}}_{\text{i}}$$

where the risk factors of interest are age at examination, sex (1 = male, 2 = female), smoking status (1 = smoker, 0 = nonsmoker), and a vector of SNPs identified in the first step. The interaction between age and sex was also considered in the model.

The analysis of correlated traits was based on GEEs [5], using up to 3 visits per subject.

#### Conditional model (subject-specific model)

Another way to accommodate the correlation arising among repeated outcomes over time is by introducing subject-specific random effects. As a result of the nested structure of the data where individuals were repeatedly measured over time and nested within families, we considered the 3-level logistic random intercepts model for the binary hypertension y_ijk _at time point *i*, of individual *j*, within family *k*, as

(3) $$\text{logitP(}{\text{y}}_{\text{ijk}}=\text{1};\;{\text{X}}_{\text{ijk}}\text{)}=\beta_{0}+{\nu_{\text{jk}}}^{\left(\text{2}\right)}+{\nu_{\text{k}}}^{\left(\text{3}\right)}+\;\beta_{\text{1}}\text{ag}{\text{e}}_{\text{ijk}}+\beta_{\text{2}}\text{se}{\text{x}}_{\text{jk}}+\beta_{\text{3}}\;\text{se}{\text{x}}_{\text{jk}}\text{x ag}{\text{e}}_{\text{ijk}}+\beta_{\text{4}}\;\text{smok}{\text{e}}_{\text{ijk}}+\;\beta_{\text{5}}\;\text{SN}{\text{P}}_{\text{jk}},$$

where ν_jk_^(2) ^represents a random effect for individual *j *within family *k *following iid N(0, σ_(2)_^2^) and ν_k_^(3) ^is a random effect for family *k *with iid N(0, σ_(3)_^2^). The two random intercepts describe the dependence at two levels: one at the individual level among repeated outcomes and another at the family level among family members.

## Results

Our analysis focused on a binary trait of hypertension defined by SBP >140 mm Hg, DBP >90 mm Hg, or use of antihypertensive medication. A total of 932 participants from 20 families were considered. The genome-wide association analyses were performed for 65,519 SNPs on chromosome 3 using the software PLINK [7] with R-plugin, and statistical analysis was conducted using SAS [8] and R [9].

## Data preprocessing

After the data were preprocessed for quality control, 850 individuals were kept with no missing phenotypes and 6261 SNPs were excluded with Hardy-Weinberg equilibrium test *p *value (among founders only) <0.001, or minor allele frequency <0.01, or with a missing genotyping rate >0.95 from our analyses.

## SNP selection

With adjustment for covariates and familial correlation, we identified the top 5 SNPs on chromosome 3 that were most associated with hypertension binary trait using the logistic model and with age at first hypertension using the Cox PH model, respectively (Tables 1 and 2). We found that the two most significant SNPs are rs10510257 and rs1047115. Interestingly, these two SNPs were identified from both models.

**Table 1:** Top 5 most significant SNPs associated with time to hypertension based on Cox PH model with frailty

Chr	SNP	Basepair position	*p *Value	In/near gene (within 60 kb)
3	rs10510257	3346138	1.09080 × 10^−5^	
3	rs1047115	186358366	1.38323 × 10^−5^	*FETUB*
3	rs5024851	247473	1.67662 × 10^−5^	*CHL1*
3	rs7630698	189199930	2.19678 × 10^−5^	
3	rs704903	43070847	4.09467 × 10^−5^	*FAM198A*

**Table 2:** Top 5 most significant SNPs associated with any occurrence of hypertension over the study period based on logistic random effect model

Chr	SNP	Basepair position	*p *value	In/near gene (within 60 kb)
3	rs10510257	3346138	6.95197 × 10^−6^	
3	rs1047115	186358366	1.73097 × 10^−5^	*FETUB*
3	rs719318	10474137	1.89300 × 10^−5^	*ATP2B2*
3	rs6807497	67015910	2.31716 × 10^−5^	
3	rs1456217	66959472	3.16978 × 10^−5^	

## Marginal and conditional models for longitudinal data

**Figure 1:**
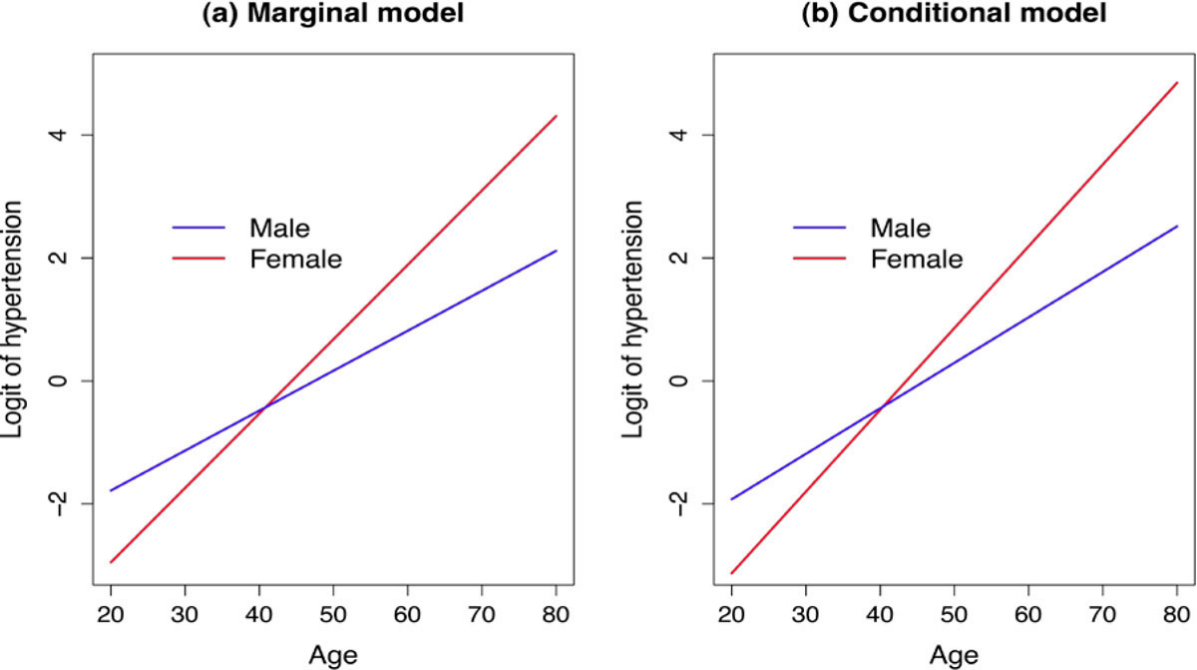
**Interaction effect between age and gender on hypertension in the marginal and conditional models**.

We compared the two approaches for modeling longitudinal binary data by including the two identified SNPs. For a fair comparison of these two models, we used only 443 individuals who completed the first 3 follow-ups with no missing genotypes in the two SNPs. Table 3 summarizes the estimates of regression coefficients and variance of random effects for marginal and conditional models. A first-order autoregressive correlation structure (AR1) was chosen for GEE because it best described the correlation structure of the data among other correlation structures. In particular, the first-order autoregressive moving average correlation structure provided almost the same results as AR1. For both models, we found that age effect on the odds of hypertension was significantly different for males and females, as shown in Figure 1. In the marginal model, females had increased odds for hypertension by 87% for each 5-year increase in age, whereas males had increased odds of only 56% for each 5-year increase in age. The genetic effects of both SNPs (rs10510257 and rs1047115) were significant with adjustment of other covariates at the 0.1 level of significance. For rs10510257, genotypes AA and AG decreased the odds of hypertension by 33% and 41%, respectively, compared to genotype GG; for rs1047115, genotype GT had a two-fold increased odds of hypertension compared to genotype TT.

**Table 3:** Comparison of marginal and conditional models: estimated coefficients from the two models for the longitudinal binary hypertension traits

	Marginal model			Conditional model		
		
Variables	Log OR	SE	*p *value	Log OR	SE	*p *value
Intercept	−4.002	1.281	0.0018	−5.012	1.618	-
Age (years)	0.053	0.026	0.0429	0.072	0.032	0.0274
Gender	−1.209	0.811	0.1359	−1.206	0.964	0.2112
Smoke	0.203	0.253	0.4211	0.213	0.315	0.5001
Age × gender	0.036	0.017	0.0331	0.036	0.019	0.0621
Rs10510257(AA)	−1.097	0.574	0.0558	−1.399	0.747	0.0615
Rs10510257(AG)	−0.887	0.286	0.0019	−1.119	0.313	0.0004
Rs1047115 (GT)	0.712	0.431	0.0985	0.903	0.536	0.0925

Random effects						

σ_(2) _for ID				1.388		
σ_(3) _for PEDNUM				1.092		

We observed that the estimates of the regression coefficients in the conditional analysis were slightly farther away from zero than those from the marginal model. For example, the log odds ratio (OR) of hypertension between smokers and nonsmokers was 0.213 in the conditional model compared to 0.203 in the marginal model. The log OR of rs10510257 genotype AA compared to genotype GG was −1.399 for the conditional model and −1.097 for the marginal model. In addition, the conditional model allowed us to measure intraclass correlations via the variances of random effects. The estimated variance of individual-level random effect was 1.388 and that of family-level random effect was 1.092. They yielded the estimated intraclass correlation across repeated measures of hypertension in the same individual equal to 0.43 and familial correlation equal to 0.19, which implies that hypertension is substantially correlated with previous experience of hypertension.

## Prediction ability

**Figure 2:**
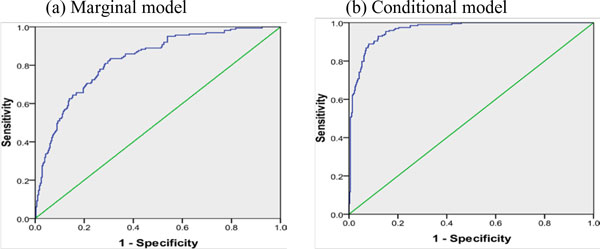
**Receiver operating characteristic curves for marginal and conditional modeling approaches**.

We demonstrated the prediction ability of the two models in Figure 2 using the receiver operating characteristic curve by predicting the hypertension status of individuals at follow-up 3. The estimates of the area under the curve (AUC) were 0.839 and 0.973 based on the marginal and conditional models, respectively, indicating that the conditional model had better ability for prediction. To compare the predictive ability of the SNPs, we obtained the AUCs for the two models without SNPs; their AUCs were, respectively, 0.826 and 0.973 for the marginal and conditional models. However, we did not see much noticeable difference in AUCs with SNPs and without SNPs for both marginal and conditional models. The increase of 0.013 in AUCs for the marginal model with SNPs compared to that without SNPs might still be suggestive of a meaningful improvement [10]. For further comparison, we obtained the correct classification rates using an arbitrary cutoff of 0.5. Although the magnitude of their increase appears relatively small, there was some improvement in the correct classification rates when the SNPs were added to the model (78% with SNPs and 76.9% without SNPs in the marginal models, and 91.5% with SNPs and 91% without SNPs in the conditional models). Indeed, the SNPs contributed more in the marginal model than in the conditional model.

## Discussions

In the analysis of longitudinal family data for genetic association studies, the correlation structure of the data is often complex and requires adequate modeling. In addition, population-average effect estimates are smaller in magnitude than subject-specific effect estimates with the difference increasing with intraclass correlation, as our analyses revealed [11].

It is worth mentioning that in our conditional model, multiple random effects were included to address the nested structure of the data where individuals are repeatedly measured over time and nested within families; in particular, we assumed a common random effect shared within families to explain familial correlation induced by shared latent environmental and genetic risk factors. However, using a single random effect may not be sufficient if we want to describe more complex correlation structure within families. The relativeness within families can be explicitly modeled using multivariate random variables or using kinship coefficient.

In our modeling of longitudinal data, although the SNPs identified were significant risk factors in the model, the improvement of predicting capability of the model measured by the AUCs appeared to be very minor yet not negligible. Notice that our genome scan was only on chromosome 3. As one reviewer pointed out, a comprehensive predictive model would not be possible without whole-genome exploration.

## Conclusion

Our study demonstrated that the genetic information plays an important role in predicting future hypertension event. In our modeling of longitudinal data, the SNPs identified were significant risk factors in the model whereas the improvement of predicting capability of the model measured by the AUCs appeared to be very minor yet not negligible. We found that the conditional modeling approach could make maximum use of the information provided by repeated phenotypes, which could lead to better prediction. Therefore, the longitudinal modeling approaches also can be helpful for identifying new genes and developing new treatments for repeated outcomes.

## Competing interests

The authors declare that they have no competing interests.

## Authors' contributions

YC designed the overall study and drafted the manuscript. BS and YC carried out the genome-wide association study; RC and YC designed the study and conducted the statistical analyses. All authors read and approved the final manuscript.
